# 1-(2-Chloro­benzo­yl)-3-(2-trifluoro­methyl­phen­yl)thio­urea

**DOI:** 10.1107/S1600536812048829

**Published:** 2012-12-05

**Authors:** M. Khawar Rauf, Masahiro Ebihara, Amin Badshah

**Affiliations:** a Department of Chemistry, Quaid-i-Azam University, Islamabad 45320, Pakistan; b Department of Chemistry, Faculty of Engineering, Gifu University Yanagido, Gifu 501-1193, Japan

## Abstract

The dihedral angle between the benzene rings in the title compound, C_15_H_10_ClF_3_N_2_OS, is 54.02 (4)°. An intra­molecular N—H⋯O hydrogen bond occurs. In the crystal, N—H⋯S hydrogen bonds link the mol­ecules into inversion dimers.

## Related literature

For our previous work on the structural and coordination chemistry of *N*,*N′*-disubstituted thio­ureas and a related structure, see: Rauf *et al.* (2012 ▶). For a description of the Cambridge Structural Database, see: Allen *et al.* (2002 ▶).
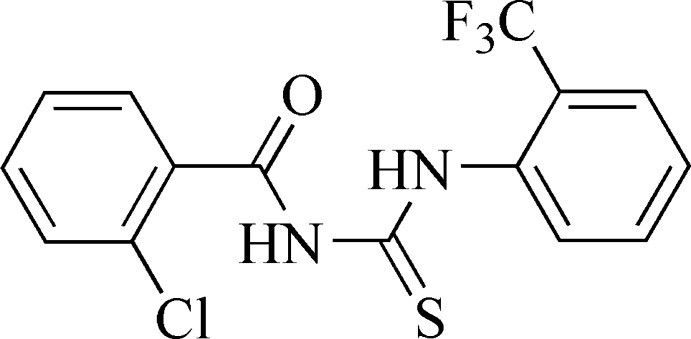


## Experimental

### 

#### Crystal data

C_15_H_10_ClF_3_N_2_OS
*M* *_r_* = 358.76
Triclinic, 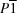

*a* = 7.705 (3) Å
*b* = 8.348 (3) Å
*c* = 12.465 (5) Å
α = 84.92 (1)°
β = 72.913 (9)°
γ = 86.272 (11)°
*V* = 762.7 (5) Å^3^
*Z* = 2
Mo *K*α radiation
μ = 0.42 mm^−1^
*T* = 123 K
0.45 × 0.36 × 0.28 mm

#### Data collection

Rigaku/MSC Mercury CCD diffractometer
5989 measured reflections
3408 independent reflections
3240 reflections with *I* > 2σ(*I*)
*R*_int_ = 0.023

#### Refinement

*R*[*F* ^2^ > 2σ(*F* ^2^)] = 0.031
*wR*(*F* ^2^) = 0.075
*S* = 1.07
3408 reflections
236 parameters
H-atom parameters constrained
Δρ_max_ = 0.38 e Å^−3^
Δρ_min_ = −0.21 e Å^−3^

### 

Data collection: *CrystalClear* (Molecular Structure Corporation & Rigaku, 2001 ▶); cell refinement: *CrystalClear*; data reduction: *CrystalClear*; program(s) used to solve structure: *SIR97* (Altomare *et al.*, 1999 ▶); program(s) used to refine structure: *SHELXL97* (Sheldrick, 2008 ▶); molecular graphics: *ORTEPII* (Johnson, 1976 ▶); software used to prepare material for publication: *Yadokari-XG* (Wakita, 2001 ▶; Kabuto *et al.*, 2009 ▶).

## Supplementary Material

Crystal structure: contains datablock(s) I, global. DOI: 10.1107/S1600536812048829/hg5276sup1.cif

Structure factors: contains datablock(s) I. DOI: 10.1107/S1600536812048829/hg5276Isup2.hkl

Supplementary material file. DOI: 10.1107/S1600536812048829/hg5276Isup3.cml

Additional supplementary materials:  crystallographic information; 3D view; checkCIF report

## Figures and Tables

**Table 1:** Hydrogen-bond geometry (Å, °)

*D*—H⋯*A*	*D*—H	H⋯*A*	*D*⋯*A*	*D*—H⋯*A*
N1—H1⋯S1^i^	0.88	2.58	3.4032 (16)	157
N2—H2⋯O1	0.88	1.91	2.6179 (16)	136

Symmetry code: (i) 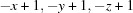.

**Fig. 1.**
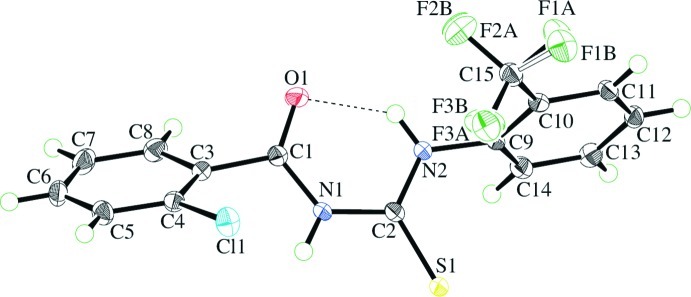
ORTEP of (I). Displacement ellipsoids are drawn at the 50% probability level. Hydrogen bonds shown as dashed lines.

**Fig. 2.**
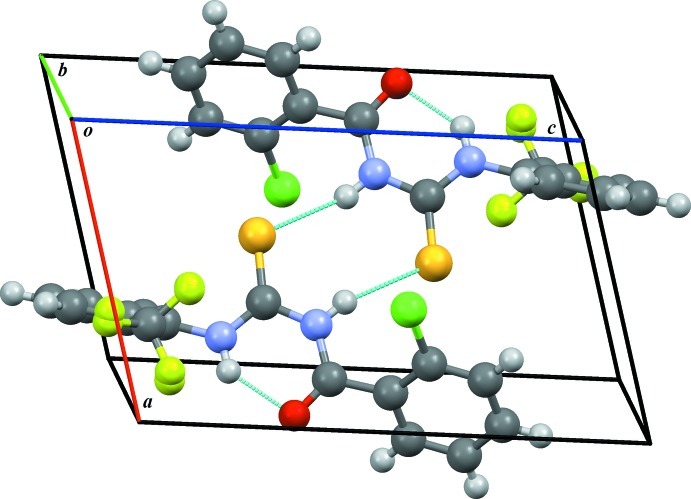
Packing diagram of (I). Hydrogen bonds shown as dashed lines.

## supplementary crystallographic information

### Comment

The background to this study has been set out in our previous work for the structural and coordination chemistry of *N*,*N'*-disubstituted thioureas (Rauf *et al.*, 2012). Herein, as a continuation of these crytallographic studies, the structure of the title compound (I) is described, Fig. 1. Compared to *N*-benzoyl-*N'*-phenylthioureas [Cambridge Structural Database (*Mogul* Version 1.7; Allen, 2002], the the trifluoromethyl moiety at C(10), implies no significant effect on these bond lengths and show the molecule to exist in the thione form with typical thiourea C—S and C—O bonds, as well as shortened C—N bond lengths. The thiocarbonyl and carbonyl groups are almost coplanar, as reflected by the torsion angles C(2)—N(1)—C(1)—O(1) [0.3 (2)] and N(2)—C(2)—N(1)—C(1) [2.0 (2)]. This is associated with the expected typical thiourea intramolecular N—H···O hydrogen bond, forming a six-membered ring commonly observed in this class of compounds (Rauf *et al.*, 2012). In the crystal packing of (I), intermolecular N—H···S H–bonds link the molecules into centrosymmetric dimers (Fig.2).

### Experimental

Freshly prepared 2-chlorobenzoylisothiocyanate (1.98 g, 10 mmol) was dissolved in tetrahydrofuran (35 ml) and stirred for 40 minutes. Afterwards neat 2-trifluoromethylaniline (1.61 g, 10 mmol) was added and the resulting mixture was stirred for 1 h. The reaction mixture was then poured into acidified water and stirred well. The solid product was separated and washed with deionized water and purified by recrystallization from chloroform to give crystals of the title compound (I), with an overall yield of 94% (3.4 g). M.P. 156–157°C Anal. calcd. for C_15_H_10_ClF_3_N_2_OS; C, 50.22 H, 2.81 N, 7.81 S, 8.94% Found: C, 50.20 H, 2.80 N, 7.81 S, 8.93%.

### Refinement

The F atoms of the trifluoromethyl group are disordered over two sites with a site occupation factor of 0.52 (9):0.48 (9) for the major and minor occupied sites respectively and were refined isotropically. Hydrogen atoms were included in calculated positions and refined as riding on their parent atom with N—H = 0.88 Å and *U*_iso_(H) = 1.2U(N_eq_), C_aromatic_—H = 0.95 Å and *U*_iso_(H) = 1.2U(C_eq_).

### Crystal data

**Table:**

C_15_H_10_ClF_3_N_2_OS	*Z* = 2
*M**_r_* = 358.76	*F*(000) = 364
Triclinic, *P*1	*D*_x_ = 1.562 Mg m^−^^3^
Hall symbol: -P 1	Mo *K*α radiation, λ = 0.71070 Å
*a* = 7.705 (3) Å	Cell parameters from 2652 reflections
*b* = 8.348 (3) Å	θ = 3.1–27.5°
*c* = 12.465 (5) Å	µ = 0.42 mm^−^^1^
α = 84.92 (1)°	*T* = 123 K
β = 72.913 (9)°	Block, colorless
γ = 86.272 (11)°	0.45 × 0.36 × 0.28 mm
*V* = 762.7 (5) Å^3^	

### Data collection

**Table:**

Rigaku/MSC Mercury CCD diffractometer	3240 reflections with *I* > 2σ(*I*)
Radiation source: Rotating Anode	*R*_int_ = 0.023
Graphite Monochromator monochromator	θ_max_ = 27.5°, θ_min_ = 3.1°
Detector resolution: 14.62 pixels mm^-1^	*h* = −8→10
ω scans	*k* = −10→10
5989 measured reflections	*l* = −16→12
3408 independent reflections	

### Refinement

**Table:**

Refinement on *F*^2^	Primary atom site location: structure-invariant direct methods
Least-squares matrix: full	Secondary atom site location: difference Fourier map
*R*[*F*^2^ > 2σ(*F*^2^)] = 0.031	Hydrogen site location: inferred from neighbouring sites
*wR*(*F*^2^) = 0.075	H-atom parameters constrained
*S* = 1.07	*w* = 1/[σ^2^(*F*_o_^2^) + (0.0273*P*)^2^ + 0.4772*P*] where *P* = (*F*_o_^2^ + 2*F*_c_^2^)/3
3408 reflections	(Δ/σ)_max_ = 0.001
236 parameters	Δρ_max_ = 0.38 e Å^−^^3^
0 restraints	Δρ_min_ = −0.21 e Å^−^^3^

### Special details

**Table:**

Geometry. All e.s.d.'s (except the e.s.d. in the dihedral angle between two l.s. planes) are estimated using the full covariance matrix. The cell e.s.d.'s are taken into account individually in the estimation of e.s.d.'s in distances, angles and torsion angles; correlations between e.s.d.'s in cell parameters are only used when they are defined by crystal symmetry. An approximate (isotropic) treatment of cell e.s.d.'s is used for estimating e.s.d.'s involving l.s. planes.
Refinement. Refinement of *F*^2^ against ALL reflections. The weighted *R*-factor *wR* and goodness of fit *S* are based on *F*^2^, conventional *R*-factors *R* are based on *F*, with *F* set to zero for negative *F*^2^. The threshold expression of *F*^2^ > σ(*F*^2^) is used only for calculating *R*-factors(gt) *etc*. and is not relevant to the choice of reflections for refinement. *R*-factors based on *F*^2^ are statistically about twice as large as those based on *F*, and *R*- factors based on ALL data will be even larger.

### Fractional atomic coordinates and isotropic or equivalent isotropic displacement parameters (Å^2^)

**Table:**

	*x*	*y*	*z*	*U*_iso_*/*U*_eq_	Occ. (<1)
C1	0.88846 (18)	0.27682 (16)	0.40388 (11)	0.0167 (3)	
O1	1.00871 (14)	0.25013 (13)	0.31743 (8)	0.0241 (2)	
N1	0.72626 (15)	0.35754 (14)	0.40451 (9)	0.0171 (2)	
H1	0.6488	0.3712	0.4711	0.020*	
C2	0.67122 (17)	0.41979 (16)	0.31202 (11)	0.0157 (2)	
S1	0.47107 (4)	0.52082 (4)	0.33205 (3)	0.01981 (10)	
N2	0.78777 (15)	0.39383 (14)	0.21154 (9)	0.0181 (2)	
H2	0.8874	0.3349	0.2097	0.022*	
C3	0.91513 (17)	0.22916 (16)	0.51696 (11)	0.0161 (3)	
C4	0.79470 (18)	0.13899 (16)	0.60350 (11)	0.0166 (3)	
C5	0.8315 (2)	0.09787 (17)	0.70526 (12)	0.0215 (3)	
H5	0.7486	0.0367	0.7637	0.026*	
C6	0.9904 (2)	0.14674 (18)	0.72090 (13)	0.0243 (3)	
H6	1.0150	0.1206	0.7909	0.029*	
C7	1.1135 (2)	0.23331 (19)	0.63536 (13)	0.0255 (3)	
H7	1.2228	0.2652	0.6463	0.031*	
C8	1.07667 (19)	0.27344 (18)	0.53359 (12)	0.0219 (3)	
H8	1.1621	0.3316	0.4746	0.026*	
Cl1	0.60119 (5)	0.06558 (4)	0.58431 (3)	0.02446 (10)	
C9	0.76060 (17)	0.45602 (16)	0.10645 (10)	0.0157 (3)	
C10	0.72840 (17)	0.35187 (16)	0.03331 (11)	0.0168 (3)	
C11	0.71224 (18)	0.41280 (17)	−0.07151 (11)	0.0182 (3)	
H11	0.6902	0.3424	−0.1215	0.022*	
C12	0.72833 (18)	0.57592 (17)	−0.10270 (11)	0.0194 (3)	
H12	0.7174	0.6172	−0.1740	0.023*	
C13	0.76050 (19)	0.67912 (17)	−0.02973 (12)	0.0211 (3)	
H13	0.7715	0.7909	−0.0513	0.025*	
C14	0.77660 (19)	0.61922 (17)	0.07490 (12)	0.0199 (3)	
H14	0.7985	0.6901	0.1247	0.024*	
C15	0.7096 (2)	0.17576 (17)	0.06715 (12)	0.0221 (3)	
F1B	0.655 (4)	0.094 (4)	−0.007 (3)	0.031 (2)	0.48 (9)
F2B	0.879 (4)	0.112 (3)	0.082 (2)	0.027 (2)	0.48 (9)
F3B	0.582 (2)	0.1436 (14)	0.1620 (9)	0.040 (4)	0.48 (9)
F1A	0.690 (5)	0.096 (4)	−0.015 (3)	0.035 (3)	0.52 (9)
F2A	0.848 (3)	0.098 (3)	0.086 (2)	0.031 (2)	0.52 (9)
F3A	0.5736 (17)	0.1467 (12)	0.1633 (7)	0.030 (3)	0.52 (9)

### Atomic displacement parameters (Å^2^)

**Table:**

	*U*^11^	*U*^22^	*U*^33^	*U*^12^	*U*^13^	*U*^23^
C1	0.0175 (6)	0.0172 (6)	0.0149 (6)	0.0001 (5)	−0.0043 (5)	−0.0007 (5)
O1	0.0208 (5)	0.0306 (6)	0.0164 (5)	0.0078 (4)	−0.0008 (4)	0.0000 (4)
N1	0.0154 (5)	0.0237 (6)	0.0105 (5)	0.0035 (4)	−0.0020 (4)	−0.0019 (4)
C2	0.0169 (6)	0.0172 (6)	0.0130 (6)	−0.0007 (5)	−0.0037 (5)	−0.0028 (5)
S1	0.01603 (16)	0.02989 (19)	0.01299 (16)	0.00589 (13)	−0.00426 (12)	−0.00353 (13)
N2	0.0174 (5)	0.0241 (6)	0.0116 (5)	0.0057 (4)	−0.0037 (4)	−0.0019 (4)
C3	0.0164 (6)	0.0168 (6)	0.0152 (6)	0.0026 (5)	−0.0053 (5)	−0.0019 (5)
C4	0.0178 (6)	0.0153 (6)	0.0173 (6)	0.0000 (5)	−0.0059 (5)	−0.0022 (5)
C5	0.0281 (7)	0.0189 (7)	0.0170 (6)	0.0004 (5)	−0.0069 (5)	0.0015 (5)
C6	0.0318 (8)	0.0236 (7)	0.0220 (7)	0.0063 (6)	−0.0160 (6)	−0.0032 (6)
C7	0.0213 (7)	0.0282 (8)	0.0321 (8)	0.0022 (6)	−0.0151 (6)	−0.0059 (6)
C8	0.0173 (6)	0.0237 (7)	0.0242 (7)	−0.0004 (5)	−0.0056 (5)	−0.0010 (5)
Cl1	0.02307 (17)	0.02779 (19)	0.02433 (18)	−0.00967 (13)	−0.00880 (13)	0.00143 (13)
C9	0.0136 (6)	0.0212 (6)	0.0105 (6)	0.0020 (5)	−0.0012 (4)	−0.0017 (5)
C10	0.0150 (6)	0.0195 (6)	0.0140 (6)	0.0006 (5)	−0.0017 (5)	−0.0015 (5)
C11	0.0170 (6)	0.0239 (7)	0.0134 (6)	−0.0004 (5)	−0.0033 (5)	−0.0032 (5)
C12	0.0156 (6)	0.0273 (7)	0.0136 (6)	0.0001 (5)	−0.0030 (5)	0.0028 (5)
C13	0.0207 (6)	0.0196 (7)	0.0214 (7)	−0.0017 (5)	−0.0044 (5)	0.0028 (5)
C14	0.0208 (6)	0.0209 (7)	0.0182 (6)	−0.0013 (5)	−0.0052 (5)	−0.0037 (5)
C15	0.0263 (7)	0.0209 (7)	0.0181 (7)	0.0001 (5)	−0.0051 (6)	−0.0010 (5)
F1B	0.043 (6)	0.020 (2)	0.034 (6)	−0.010 (4)	−0.015 (5)	−0.005 (3)
F2B	0.021 (5)	0.016 (3)	0.042 (2)	0.003 (3)	−0.011 (3)	0.003 (2)
F3B	0.046 (6)	0.023 (5)	0.042 (6)	−0.003 (3)	0.004 (4)	−0.004 (4)
F1A	0.054 (9)	0.025 (2)	0.029 (3)	−0.010 (5)	−0.014 (6)	−0.0042 (18)
F2A	0.023 (5)	0.024 (3)	0.049 (3)	0.001 (3)	−0.013 (3)	0.000 (2)
F3A	0.032 (4)	0.029 (5)	0.019 (4)	−0.009 (3)	0.002 (3)	0.014 (3)

### Geometric parameters (Å, º)

**Table:**

C1—O1	1.2240 (17)	C8—H8	0.9500
C1—N1	1.3790 (17)	C9—C14	1.387 (2)
C1—C3	1.4992 (18)	C9—C10	1.3964 (19)
N1—C2	1.3898 (17)	C10—C11	1.3968 (19)
N1—H1	0.8800	C10—C15	1.497 (2)
C2—N2	1.3356 (17)	C11—C12	1.386 (2)
C2—S1	1.6714 (14)	C11—H11	0.9500
N2—C9	1.4338 (17)	C12—C13	1.389 (2)
N2—H2	0.8800	C12—H12	0.9500
C3—C4	1.3964 (19)	C13—C14	1.392 (2)
C3—C8	1.3978 (19)	C13—H13	0.9500
C4—C5	1.3889 (19)	C14—H14	0.9500
C4—Cl1	1.7350 (14)	C15—F2A	1.28 (2)
C5—C6	1.386 (2)	C15—F3B	1.317 (13)
C5—H5	0.9500	C15—F1A	1.32 (3)
C6—C7	1.384 (2)	C15—F3A	1.355 (10)
C6—H6	0.9500	C15—F1B	1.37 (3)
C7—C8	1.388 (2)	C15—F2B	1.43 (2)
C7—H7	0.9500		
			
O1—C1—N1	123.21 (12)	C9—C10—C11	119.71 (13)
O1—C1—C3	120.74 (12)	C9—C10—C15	120.33 (12)
N1—C1—C3	115.98 (11)	C11—C10—C15	119.96 (12)
C1—N1—C2	127.40 (11)	C12—C11—C10	120.07 (13)
C1—N1—H1	116.3	C12—C11—H11	120.0
C2—N1—H1	116.3	C10—C11—H11	120.0
N2—C2—N1	115.63 (12)	C11—C12—C13	120.04 (13)
N2—C2—S1	124.81 (10)	C11—C12—H12	120.0
N1—C2—S1	119.55 (10)	C13—C12—H12	120.0
C2—N2—C9	124.05 (11)	C12—C13—C14	120.19 (13)
C2—N2—H2	118.0	C12—C13—H13	119.9
C9—N2—H2	118.0	C14—C13—H13	119.9
C4—C3—C8	118.57 (12)	C9—C14—C13	119.96 (13)
C4—C3—C1	124.71 (12)	C9—C14—H14	120.0
C8—C3—C1	116.66 (12)	C13—C14—H14	120.0
C5—C4—C3	120.93 (13)	F2A—C15—F3B	101.9 (12)
C5—C4—Cl1	118.00 (11)	F2A—C15—F1A	100 (2)
C3—C4—Cl1	120.97 (10)	F3B—C15—F1A	111.6 (15)
C6—C5—C4	119.47 (13)	F2A—C15—F3A	104.1 (12)
C6—C5—H5	120.3	F1A—C15—F3A	111.2 (15)
C4—C5—H5	120.3	F2A—C15—F1B	107.3 (18)
C7—C6—C5	120.53 (13)	F3B—C15—F1B	101.8 (14)
C7—C6—H6	119.7	F3A—C15—F1B	101.3 (13)
C5—C6—H6	119.7	F3B—C15—F2B	107.7 (11)
C6—C7—C8	119.83 (14)	F1A—C15—F2B	104 (2)
C6—C7—H7	120.1	F3A—C15—F2B	109.9 (10)
C8—C7—H7	120.1	F1B—C15—F2B	112.2 (17)
C7—C8—C3	120.63 (13)	F2A—C15—C10	117.1 (11)
C7—C8—H8	119.7	F3B—C15—C10	113.8 (6)
C3—C8—H8	119.7	F1A—C15—C10	111.2 (14)
C14—C9—C10	120.03 (12)	F3A—C15—C10	112.3 (5)
C14—C9—N2	119.62 (12)	F1B—C15—C10	113.3 (14)
C10—C9—N2	120.25 (12)	F2B—C15—C10	107.9 (10)
			
O1—C1—N1—C2	0.3 (2)	C14—C9—C10—C11	−0.06 (19)
C3—C1—N1—C2	177.38 (12)	N2—C9—C10—C11	−176.44 (11)
C1—N1—C2—N2	2.0 (2)	C14—C9—C10—C15	−179.55 (12)
C1—N1—C2—S1	−177.43 (11)	N2—C9—C10—C15	4.08 (19)
N1—C2—N2—C9	−176.33 (12)	C9—C10—C11—C12	0.07 (19)
S1—C2—N2—C9	3.1 (2)	C15—C10—C11—C12	179.56 (12)
O1—C1—C3—C4	−128.44 (15)	C10—C11—C12—C13	0.0 (2)
N1—C1—C3—C4	54.45 (18)	C11—C12—C13—C14	0.0 (2)
O1—C1—C3—C8	48.70 (19)	C10—C9—C14—C13	0.0 (2)
N1—C1—C3—C8	−128.41 (13)	N2—C9—C14—C13	176.40 (12)
C8—C3—C4—C5	1.8 (2)	C12—C13—C14—C9	0.0 (2)
C1—C3—C4—C5	178.93 (13)	C9—C10—C15—F2A	−62.3 (13)
C8—C3—C4—Cl1	−174.53 (10)	C11—C10—C15—F2A	118.2 (13)
C1—C3—C4—Cl1	2.57 (19)	C9—C10—C15—F3B	56.3 (7)
C3—C4—C5—C6	−0.2 (2)	C11—C10—C15—F3B	−123.2 (7)
Cl1—C4—C5—C6	176.29 (11)	C9—C10—C15—F1A	−176.6 (17)
C4—C5—C6—C7	−1.2 (2)	C11—C10—C15—F1A	3.9 (17)
C5—C6—C7—C8	0.8 (2)	C9—C10—C15—F3A	58.0 (5)
C6—C7—C8—C3	0.9 (2)	C11—C10—C15—F3A	−121.5 (5)
C4—C3—C8—C7	−2.2 (2)	C9—C10—C15—F1B	172.0 (13)
C1—C3—C8—C7	−179.51 (13)	C11—C10—C15—F1B	−7.5 (13)
C2—N2—C9—C14	71.67 (18)	C9—C10—C15—F2B	−63.2 (11)
C2—N2—C9—C10	−111.94 (15)	C11—C10—C15—F2B	117.3 (11)

### Hydrogen-bond geometry (Å, º)

**Table:**

*D*—H···*A*	*D*—H	H···*A*	*D*···*A*	*D*—H···*A*
N1—H1···S1^i^	0.88	2.58	3.4032 (16)	157
N2—H2···O1	0.88	1.91	2.6179 (16)	136

Symmetry code: (i) −*x*+1, −*y*+1, −*z*+1.
